# A reconnaissance survey of channel bank particulate phosphorus concentrations, controls and estimated contributions to riverine loads across England

**DOI:** 10.1002/hyp.14785

**Published:** 2022-12-26

**Authors:** Simon Pulley, Yusheng Zhang, Ruth Copeland‐Phillips, Atish N. Vadher, Ian D.L. Foster, John Boardman, Adrian L. Collins

**Affiliations:** ^1^ Net Zero and Resilient Farming Rothamsted Research Okehampton UK; ^2^ Faculty of Arts, Science & Technology University of Northampton Northampton UK; ^3^ Department of Geography Rhodes University Makhanda (Grahamstown) South Africa; ^4^ School of Geography and the Environment University of Oxford Oxford UK; ^5^ Department of Geography University of the Free State Bloemfontein South Africa

**Keywords:** catchment management, channel banks, phosphorus, sediment source

## Abstract

Channel banks can contribute a significant proportion of fine‐grained (<63 μm) sediment to rivers, thereby also contributing to riverine total particulate phosphorus loads. Improving water quality through better agricultural practices alone can be difficult since the contributions from non‐agricultural sources, including channel banks, can generate a ‘spatial mismatch’ between the efficacy of best management applied on farms and the likelihood of meeting environmental objectives. Our study undertook a reconnaissance survey (*n* = 76 sites each with 3 profiles sampled) to determine the total phosphorus (TP) concentrations of channel banks across England and to determine if TP content can be predicted using readily accessible secondary data. TP concentrations in adjacent field topsoils, local soil soil type/texture and geological parent material were examined as potential predictors of bank TP. Carbon and nitrogen content were also analysed to explore the impacts of organic matter content on measured TP concentrations. The results suggest that channel bank TP concentrations are primarily controlled by parent material rather than P additions to adjacent topsoils through fertilizer and organic matter inputs, but significant local variability in concentrations prevents the prediction of bank TP content using mapped soil type or geology. A median TP concentration of 873 mg kg^−1^ was calculated for the middle section of the sampled channel bank profiles, with a 25th percentile of 675 mg kg^−1^, and 75th percentile of 1159 mg kg^−1^. Using these concentrations and, in comparison with previously published estimates, the estimated number of inland WFD waterbodies in England for which channel bank erosion contributes >20% of the riverine total PP load increased from 15 to 25 (corresponding range of 17–35 using the 25th and 75th percentiles of measured TP concentrations). Collectively, these 25 waterbodies account for 0.2% of the total inland WFD waterbody area comprising England.

## Graphical abstract

The assessment and, where necessary, improved management, of particulate phosphorus loss from eroding channel banks at catchment and strategic scale is important for water quality protection and provision of ecosystem services. A reconnaissance survey of the total phosphorus content of channel banks was undertaken at national scale across England and the new information generated was employed to update estimates of the relative contributions of bank‐derived inputs to riverine total phosphorus loads at Water Framework Directive (WFD) waterbody scale.

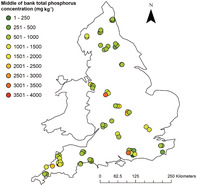

## 1 INTRODUCTION

Excess fine‐grained sediment and phosphorus (P) losses to freshwaters are associated with a reduction in water quality and concomitant decline in aquatic biodiversity (George et al., 2017; Jones et al., 2012; Rockström et al., 2009). Achieving a reduction in bioavailable soluble reactive phosphorus (SRP) is primarily targeted by existing policy in the UK; however, the dominant proportion of P load frequently enters watercourses in association with fine‐grained sediment rather than in dissolved form (Lloyd et al., 2019; Meybeck & Helmer, 1989; Royer et al., 2006). Due to the large surface area of fine‐grained sediment (Horowitz, 1991) and its substrates of chemically active Fe, Al and Ca oxyhydroxides, and humic matter, it has a high P adsorption and storage capacity, and as such, P readily exchanges between its surface and the water column (Taylor & Kunishi, 1971; Stone & Mudroch, 1989; Stone & English, 1993). Whilst point sources, such as sewage treatment works, are often major contributors to the total P load of rivers, it has been estimated that a large proportion of riverine particulate phosphorus (PP), and therefore, total P loads originate from diffuse sources such as agriculture or channel banks (Morse et al., 1993; Zhang et al., 2014). As a result, existing environmental policy and legislation specifies that reductions in P losses from both diffuse and point sources must be achieved (US Congress Clean Water Act, 1972; Water Framework Directive 2000/60/EC). With regulatory measures already in place for different sectors contributing to water pollution, achieving further reductions in riverine P concentrations and loads has become a technological and economic challenge. Catchment management strategies are therefore increasingly required to reduce the P losses to freshwaters to reach threshold target concentrations where they have been established (McDowell et al., 2016). However, establishing thresholds for freshwater PP concentrations is challenging due to spatial variability in natural background concentrations present on a national scale, and a lack of existing strategic data on the P concentrations of some key potential sources. Such an understanding is required, however, to target mitigation measures appropriately and to deliver improved benefit: cost from catchment interventions (Haygarth et al., 2009; Heathwaite et al., 2005; Kronvang et al., 2007).

Within most catchments in temperate landscapes, the erosion of agricultural topsoils is the dominant source of fine‐grained sediment, and therefore of the PP, delivered to freshwaters. However, eroding channel banks can also contribute a large proportion of riverine sediment load and therefore potentially of the total PP load (Collins et al., 1997; Collins et al., 2012; De Rose et al., 2005; Evans, 2019; Evans et al., 2003; Imeson et al., 1984; Kronvang et al., 2013; Laubel et al., 2003; Lu et al., 2015; Miller et al., 2014; Neal & Andera, 2015; Owens et al., 2000; Rode et al., 2018; Walling et al., 1999; Walling & Collins, 2005; Walling, Collins, et al., 2008; Walling, Webb, et al., 2008; Wilkinson et al., 2005; Zaimes et al., 2008a). For example, 7%–10% of the total P load of the Blue Earth River in Minnesota, USA, originated from channel bank erosion (Sekely et al., 2002) compared with up to 56% of the P load in rivers in central Illinois, USA (Roseboom, 1987). Up to 90% of the TP load of some Danish rivers has been reported to originate from bank erosion (Kronvang et al., 1997). Similarly, Walling, Webb, et al. (2008) and Walling, Collins, et al. (2008) reported that up to 43% of the PP flux in 12 agricultural sub‐catchments in the UK originated from channel bank or subsurface source erosion. On the basis of existing studies reported in the international literature, eroding channel banks have been estimated to account typically for between 10% (Sekely et al., 2002) and 40% (Howe et al., 2011) of the total P load in any individual river catchment, although this can be upwards of 90% (Kronvang et al., 1997).

Despite the potential importance of channel bank erosion as a contributor to riverine PP loads, information on the concentrations of TP characterizing this catchment source are relatively scant in published literature (Fox et al., 2016). The review by Fox et al. (2016) reported that the data which exists typically suggests that TP concentrations in bank material can exceed 250 mg kg^−1^. However, concentrations as low as 171 mg kg^−1^ were reported by Beck et al. (2018) in Iowa, USA, whilst a much wider range of concentrations spanning 130 to 1207 mg P kg^−1^ was reported by Granger et al. (2021) in the upper River Taw in southwest England. Without a reliable understanding of the concentrations of TP in channel banks, on account of their potential to be a reasonably important catchment sediment source, and of the key controlling factors on such content, reliably estimating TP loads at strategic scale for catchment management and policy support is challenging.

Failure to take explicit account of TP inputs from non‐topsoil sediment sources, will result in a ‘spatial mismatch’ between the efficacy of mitigation options targeted at farm scale and the potential impacts on riverine loads at landscape scale (Biddulph et al., 2017). Given this risk, farmers, catchment managers and policy makers need reliable information on the TP content of all potential sources to help inform the targeting of mitigation options (Glavan et al., 2012; Kronvang et al., 2012). Here, given the urgency of many policy decisions, P models are frequently used as a catchment management tool to quantify loads and associated pollution gaps requiring abatement (Radcliffe et al., 2009). However, channel bank erosion is rarely included as a sediment and PP source in catchment scale models despite empirical evidence suggesting their significance (Kronvang et al., 2012). To estimate rates of channel bank erosion to fill this scientific gap for strategic data in England, Collins and Anthony (2008) developed a channel bank erosion index based on river regime, channel density as a measure of the opportunity for bank erosion and the duration of excess shear stress as a percentage of annual river runoff. This index was evaluated using information on bank sediment yields derived from integrating suspended sediment yield data (Cooper et al., 2006) with source proportions based on sediment source fingerprinting (e.g., Walling & Collins, 2005). More recent work has employed the key physical factors controlling channel bank erosion processes, including upstream area, channel confinement and sinuosity to both evaluate and update the original index (Janes et al., 2017; Zhang et al., 2014). By relating these composite indices to mapped channel bank positions in different time periods or estimated bank erosion yields produced using sediment source apportionment procedures and either measured (e.g., Walling & Webb, 1987; Walling, Webb, et al., 2008) or extrapolated (Cooper et al., 2006) catchment suspended sediment yields, bank erosion rates in river catchments can be estimated (Janes et al., 2017). By combining the updated version of the Collins and Anthony (2008) bank erosion index with the typical TP concentrations of channel banks, Zhang et al. (2014) generated estimates of the channel bank‐derived proportions of riverine TP loads for all inland Water Framework Directive surface waterbodies (WFD waterbodies hereafter) across England and Wales. However, little data was available with which to estimate bank TP concentrations and therefore significant uncertainties were potentially associated with these initial strategic estimates.

Mapped strategic scale TP concentration data exist for topsoils in the UK and these have been used as proxy values for the TP contents of channel banks in some previous research (Deuthmann et al., 2009). Alongside the topsoil data, limited published TP content data is available as a by‐product of sediment source apportionment work where source‐specific geochemistry analysis included measurement of TP content (e.g., Walling, Webb, et al., 2008). These and additional data (Collins pers. comm.) were the basis for the quantification of WFD waterbody scale bank erosion TP contributions for England and Wales (Zhang et al., 2014). However, there is a need for more robust estimates of the TP content of eroding channel banks across England.

Against the above background, this study conducted a reconnaissance survey of TP concentrations in the channel banks of rivers across England, to provide new data on concentrations, to explore and identify controlling factors, and to determine if there is potential to predict channel bank‐associated TP concentrations using local landscape characteristics. Finally, using our new strategic scale estimates of channel bank TP content, the estimated contributions of the riverine total PP load for inland WFD waterbodies across England contributed eroding by channel banks were used to update the work of Zhang et al. (2014).

## 2 MATERIALS AND METHODS

### 2.1 Field sampling

**FIGURE 1:**
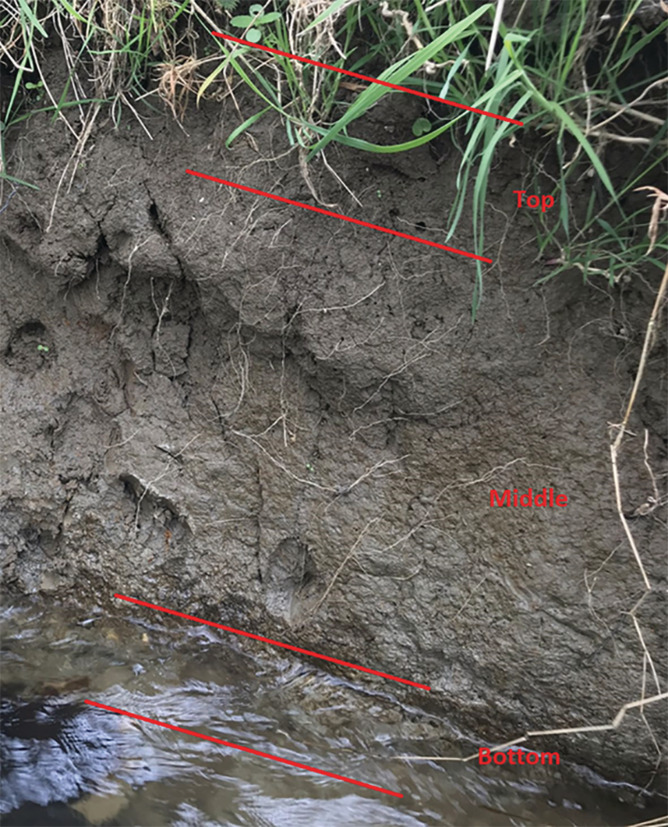
A typical channel bank with the three layers sampled highlighted

Channel bank TP concentrations were sampled at 76 sites across England. The sampling design was primarily aimed at achieving a reconnaissance level of spatial coverage at national scale. The selection of sampling sites was guided by intrinsic channel bank erosion risks and known channel margin poaching intensity; that is, areas with significant bank erosion. The former was characterized using detailed river network (DRN)‐based channel sinuosity (Zhang et al., 2014) and the latter was based on the most recent River Habitat Survey (RHS) in 2007–2008 where reach scale poaching scores were recorded (https://www.riverhabitatsurvey.org/). The final selection of a site was also inevitably dictated by its accessibility. Three channel bank profiles were sampled at each site, although this was occasionally fewer depending on the accessibility of suitable sampling locations within a 25 m radius of the chosen sampling point. Each channel bank profile was free of vegetation cover and thereby exposed to fluvial erosion. For each profile, the uppermost section where the bank material colour reflected topsoils (i.e., upper ~0–20 cm depth), bottom section where the material colour often indicated a clay layer over its parent material (i.e., lowest ~20 cm), and intervening middle section, were sampled separately (Figure 1). Three to five subsamples were taken from each section using a stainless‐steel knife and bulked together into a single bag. A sample of topsoil from the land use adjacent to the sampled channel bank, but outside of any riparian zone, was also collected from the top 5 cm of the soil profile. Dominant land uses adjacent to the banks were arable (29 sites), grassland (37 sites) and woodland (10 sites). Between all arable fields and the river channels, some form of buffer strip containing grass or trees was present, the widths of which were highly variable.

### 2.2 Laboratory analyses

Samples were oven dried at 105°C before being disaggregated using a grinding mill (mechanized pestle and mortar) and sieved to <63 μm through a stainless‐steel mesh. TP was determined by fusion with sodium hydroxide followed by colorimetric measurement using the Molybdate reactive P method as molybdenum blue (Smith & Bain, 1982). Between 0.1 and 0.25 g of the prepared sample was fused with 1¾–2½ g of sodium hydroxide in a nickel crucible over a Bunsen flame. 20 ml of ultra‐pure deionized water was added to the cooled samples and they were left to stand for 2 h. The contents of the crucible were then made up to 100 ml with ultrapure deionized water and mixed thoroughly. A 5 ml subsample was extracted and centrifuged at 3000 revolutions min^−1^ for 10 min. The supernatant was diluted to a total volume of 30 ml and adjusted to pH 6 through the addition of 2.5 M sulphuric acid. Sample TP concentration in the resultant solution was determined using a Thermo‐Fisher Aquakem 250 (Loughborough, UK) discrete photometric analyser according to the methods of Murphy and Riley (1962). The instrument has a limit of detection of 1.5 mg P l^−1^.

Sample total carbon and nitrogen concentrations were measured using a Carlo Erba NA2000 elemental analyser and a SerCon 20–22 isotope ratio mass spectrometer. Samples were packed into pure tin capsules and sealed for analysis. Wheat flour (IA‐R001 from Iso‐Analytical, Crewe, UK) (1.88% N, 40.2% C, 2.55 δ15N and −26.43 δ13C) calibrated against IAEA‐N‐1 (ammonium sulphate) for nitrogen and IAEA‐CH6 (ANU sucrose) for carbon were used for reference standards and the elemental contents were determined from the total peak areas (Dixon et al., 2010). The 95% confidence limits of analytical precision were measured at ±10% for TN and ±10.5% for TC.

### 2.3 Data analysis

Details on all sampling sites and the corresponding TP data are provided in Supplementary Table 1. The TP concentration of the middle layer of the sampled channel bank profiles was used for the data analysis as this layer made up most of the height of most bank profiles considering that the top and bottom layers only represented a height of up to 40 cm. It was also considered important to separate the effects of the topsoil, which is potentially enriched in P by agriculture in the upper section of the bank, and the naturally occurring parent material in the lower section, on the TP concentration of the majority of the bank material. A histogram of the middle section TP concentrations was used to show the distribution of concentrations within the assembled dataset. At this point, outliers were removed as they were likely to be caused by unique local factors and therefore not generally representative of channel banks.

Mean TP concentrations in the bottom, middle and top sections of the sampled channel bank profiles were compared. Correlations between the TP concentrations of the different sections of the channel bank profiles were examined. This was to identify if the concentrations in the middle section dominating the sampled channel bank profiles were primarily controlled by those in the uppermost section, which would indicate that P inputs to soils were a major control, or those in the bottom most section, indicating that parent material was the major control. The same correlations were examined but using C and N to represent organic matter as a potential control.

Within‐site variability (i.e., standard deviation) between the three channel bank profiles sampled at each site was compared to between‐site variability (standard deviation of the average TP concentrations at each sampled site). This was used to determine how much local scale variability impacts channel bank TP concentrations.

Middle channel bank profile TP concentrations at two extreme outlier sites removed from the analysis described above were compared to mapped landscape properties. These were: soil data from the GB soil map classified by texture, waterbody type (e.g., Low and Siliceous, Geology from British Geological Survey maps of Great Britain) and TP concentrations in fields adjacent to the channel bank sampling locations.

The updated channel bank TP concentration data was finally used to update the estimated proportion of the TP load of rivers across England originating from bank erosion originally reported by Zhang et al. (2014). Here, national scale inputs and contributions to riverine TP loads was estimated using the SEPARATE (SEctor Pollutant AppoRtionment for the AquaTic Environment) screening tool. This tool apportions riverine loads of TP between diffuse agricultural, diffuse urban, atmospheric deposition, sewage treatment works, septic tank, combined sewer overflow, storm tank and channel bank sources. Contributions from the latter are predicted based on a modified version of the bank erosion index originally reported by Collins and Anthony (2008), Collins et al. (2009a), and Collins et al. (2009b). This index uses river regime and duration of excess shear stress as a percentage of the flow year. Flow duration curves are generated using Gustard et al. (1992) with corresponding Q_95_ values assigned to each soil series using the hydrology of soil types (HOST) scheme (Boorman et al., 1995). Shear stress on channels banks is generated using Guo and Julien (2005) and using an assumption of a rectangular channel cross‐profile. The relationship published by Julian and Torres (2006) is used to estimate the critical shear stress threshold for channel banks across England. Channel density was also incorporated in the original index as a proxy for the opportunity for channel bank erosion. Channel bank sediment loss was calibrated using source fingerprinting data from 22 catchments (Walling & Collins, 2005). Zhang et al. (2014) updated the original index to reflect geomorphological control due to curvature. Here, the Detailed River Network (DRN; Environment Agency, UK) was used to estimate channel density and sinuosity for all inland WFD waterbodies. Regression suggested a positive relationship (*r* ^2^ = 0.66) between channel bank erosion yield and channel density multiplied by sinuosity, where the proportion of the channel network with a sinuosity between 1.3 and 1.7 exceeded 10%. Accordingly, Zhang et al. (2014) applied the original index in WFD waterbodies with <10% channel sinuosity of 1.3–1.7 and the modified index in those with >10% corresponding sinuosity. The updated index was calibrated using sediment source fingerprinting data from 30 study catchments (Collins pers. comm.).

## 3 RESULTS

### 3.1 TP concentrations in channel banks

**FIGURE 2:**
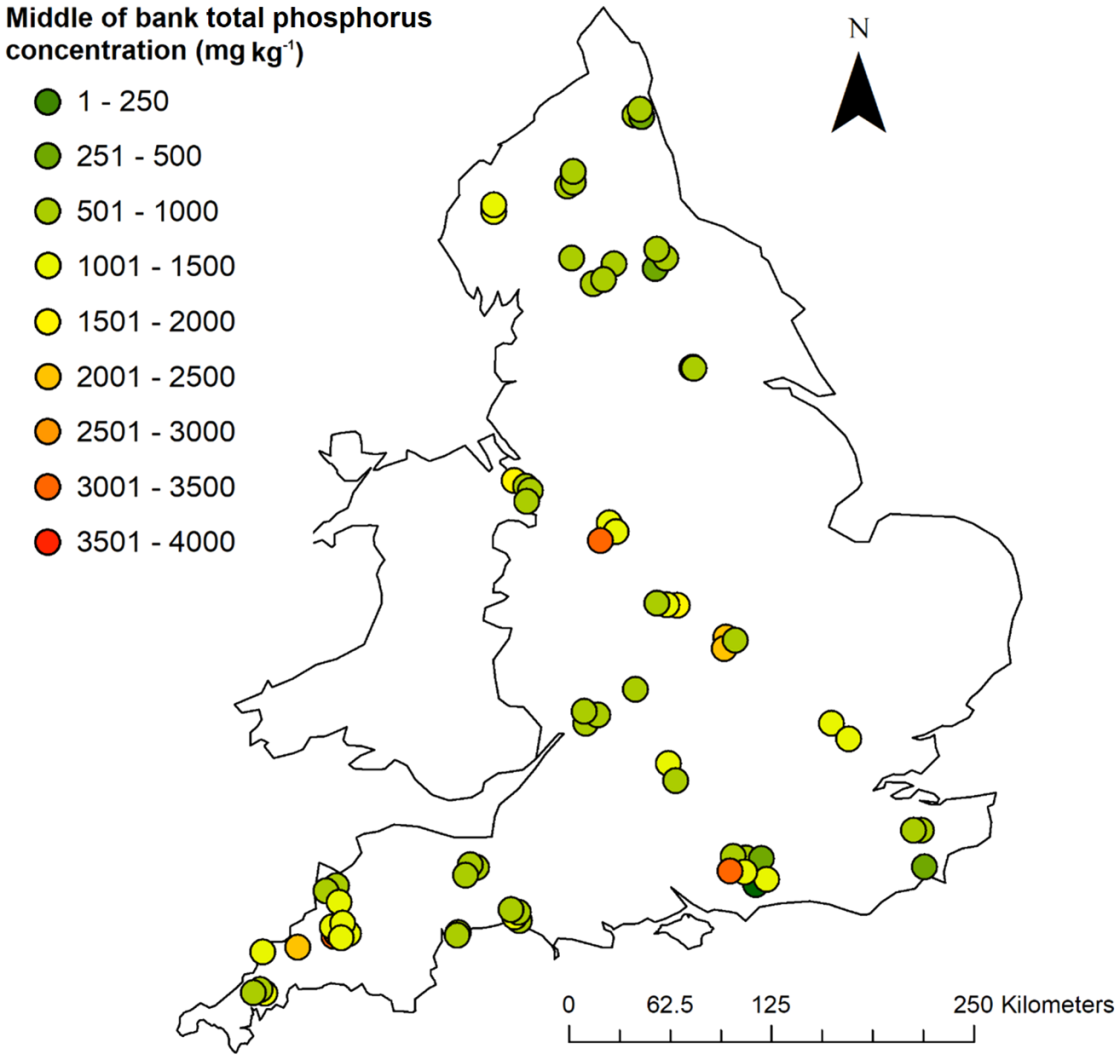
Mean TP concentrations measured in the middle sections of sampled channel bank profiles (where banks were too low to have a middle section, the values for the top and bottom sections were averaged)

**FIGURE 3:**
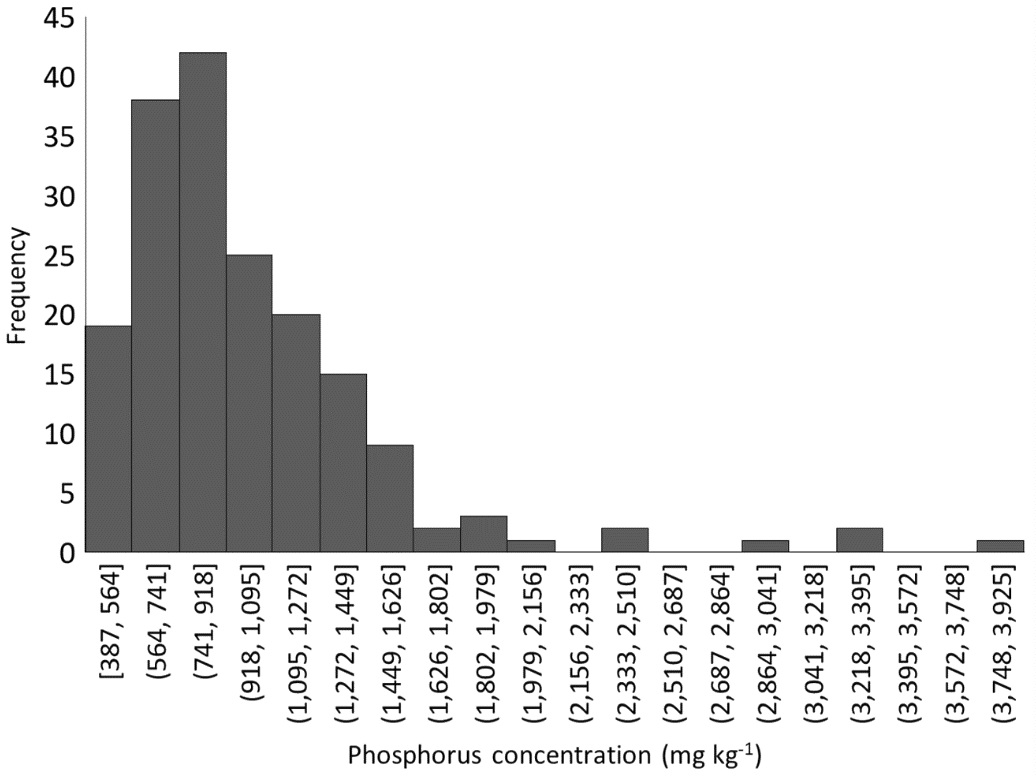
TP concentrations in the middle section of the sampled channel bank profiles

For the samples of the middle sections of the channel bank profiles (Figures 2 and 3), 93% of sampled sites had TP concentrations ranging between 387 and 1626 mg kg^−1^, with a corresponding median concentration of 873 mg kg^−1^. The remaining 7% of sites had outlying high values which most likely either reflect local geology or P‐rich topsoil or sediment which has been displaced or deposited fluvially. Of the sites with the highest TP concentrations, one was in the Nene River catchment in eastern England with a concentration of 2069 mg kg^−1^. This catchment contains an oordial ironstone geology which is naturally high in P and readily absorbs SRP from river and groundwater (Tye et al., 2016). Other sites with high TP concentrations were Stow 3 on Dagdale Brook near Bramshall (3037–3925 mg kg^−1^) in a mixed arable and grassland landscape, and Launceston 1 (2499–3336 mg kg^−1^) which is in an area of grassland where remains of a medieval ridge and furrow farming are still evident. No clear explanation for these high concentrations could be determined although the channel banks may have been reworked historically. For example, the Stow 3 site is located in close proximity to the Trent and Mersey canal, the construction of which may have involved the reworking of the river. None of the sites with high bank P concentrations had sewage treatment works (STW) in proximity upstream.

TP concentrations were generally highest in the uppermost section of most of the sampled channel bank profiles, with a corresponding mean of 98.1% of the maximum recorded in any of the three sections of any individual bank. The middle and bottom section TP concentrations were comparable and generally lower than the uppermost layer concentrations, with a corresponding mean of 85.2% of the maximum for the middle section and 85.3% for the bottom section.

### 3.2 Controls on TP concentrations in channel banks

**FIGURE 4:**
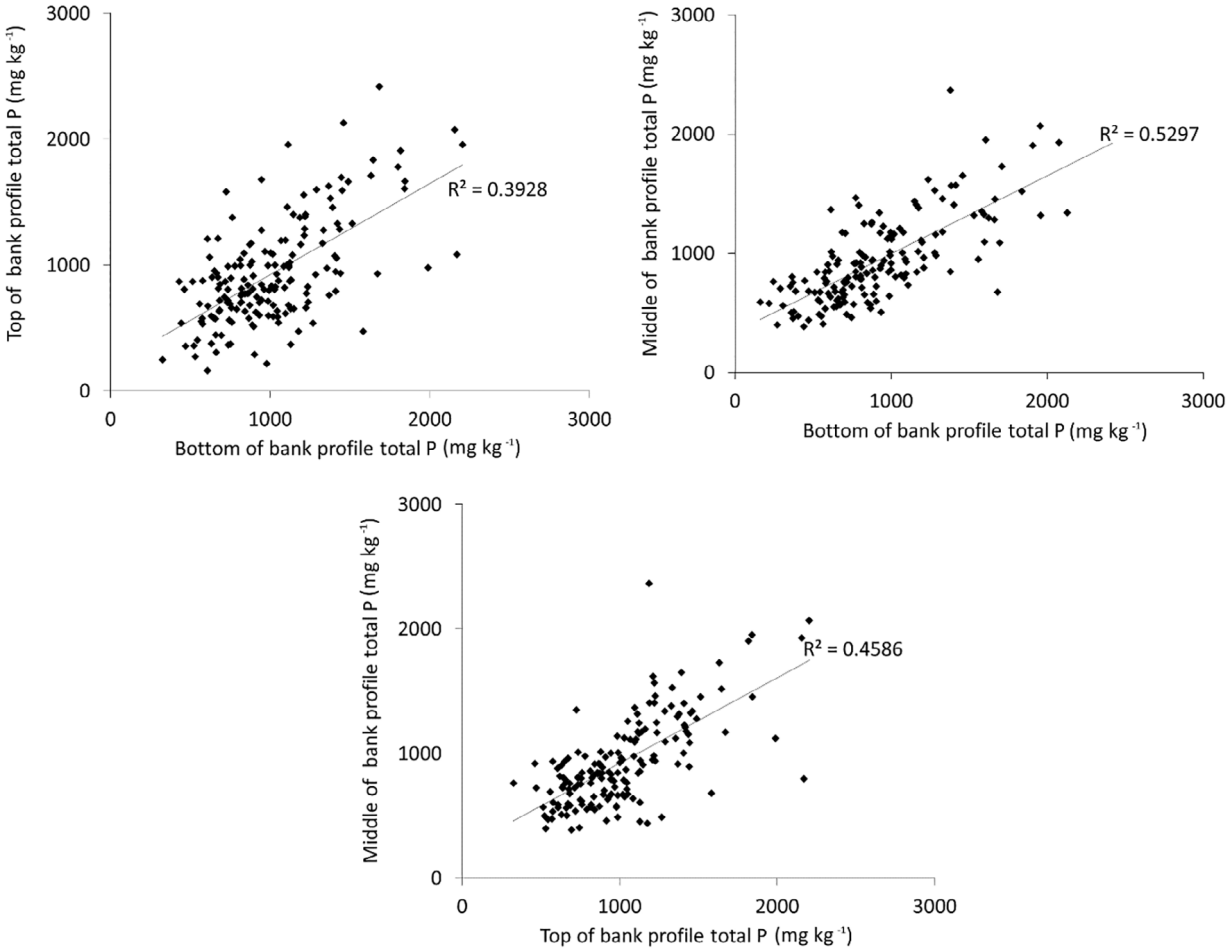
Relationships between TP concentrations in the different sections of the sampled channel bank profiles

TP concentrations in the middle section of the sampled bank profiles, were more strongly correlated with those in the bottom 20 cm of the profile than the uppermost 20 cm (Figure 4). These correlations showed a different trend for C and N. For C, there was a *r* ^2^ of 0.46 between the top and middle sections of the channel bank profiles, 0.17 between the middle and bottom and 0.11 between the bottom and uppermost sections. This suggests that C inputs from topsoils are a stronger control on the TP concentrations in the middle section of the sampled channel banks. For N, these correlations returned an *r* ^2^ of 0.36 between the top and middle sections, 0.35 between the middle and bottom and 0.20 between the bottom and uppermost sections. Therefore, the TP concentration of the middle section of the sampled channel bank profiles appears mostly controlled by the naturally occurring P concentration in its parent material rather than P enrichment moving down through the profile from toposils, whilst C and N are more significantly controlled by their concentrations in the overlying soil likely due to the presence of organic matter and fertilizer applications. There was no significant correlation between the TP concentrations in the middle sections of the channel bank profiles and the corresponding concentration in adjacent field topsoils, again confirming the negligible impact of topsoil P on channel bank P.

**FIGURE 5:**
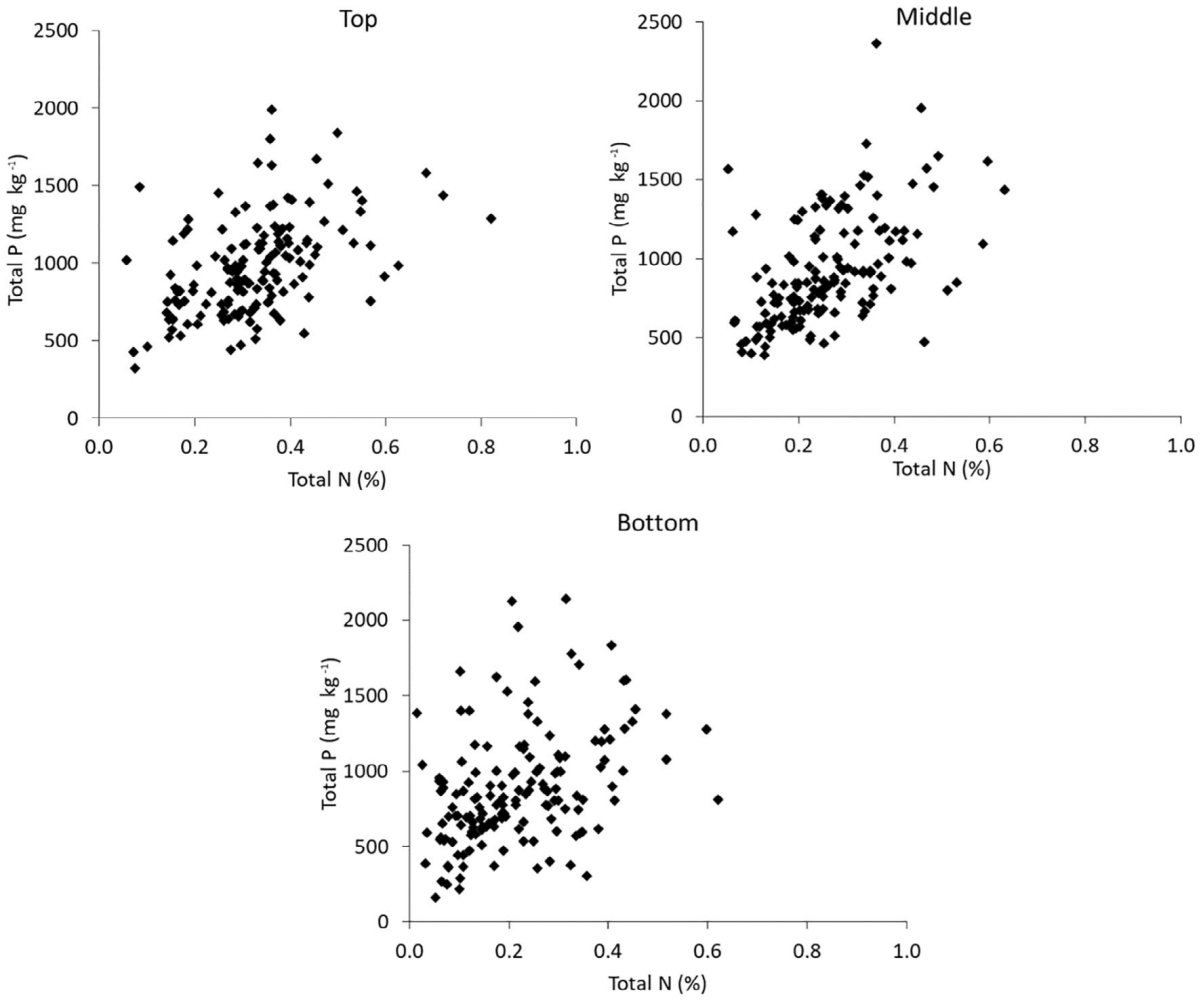
Relationships between TP and TN in the three sections of the sampled channel bank profiles

To determine the factors controlling channel bank TP concentrations, the relationships between TP and N were examined as N is likely to be primarily controlled by the amount of organic matter within the bank material. N was used, rather than C in this analysis, due to the strong correlation between the two and since a significant number of C measurements were below the LOD. There were no significant correlations between channel bank TP and N in any of the channel bank profile sections, again indicating that TP concentrations are not primarily controlled by organic matter inputs from topsoils (Figure 5).

**FIGURE 6:**
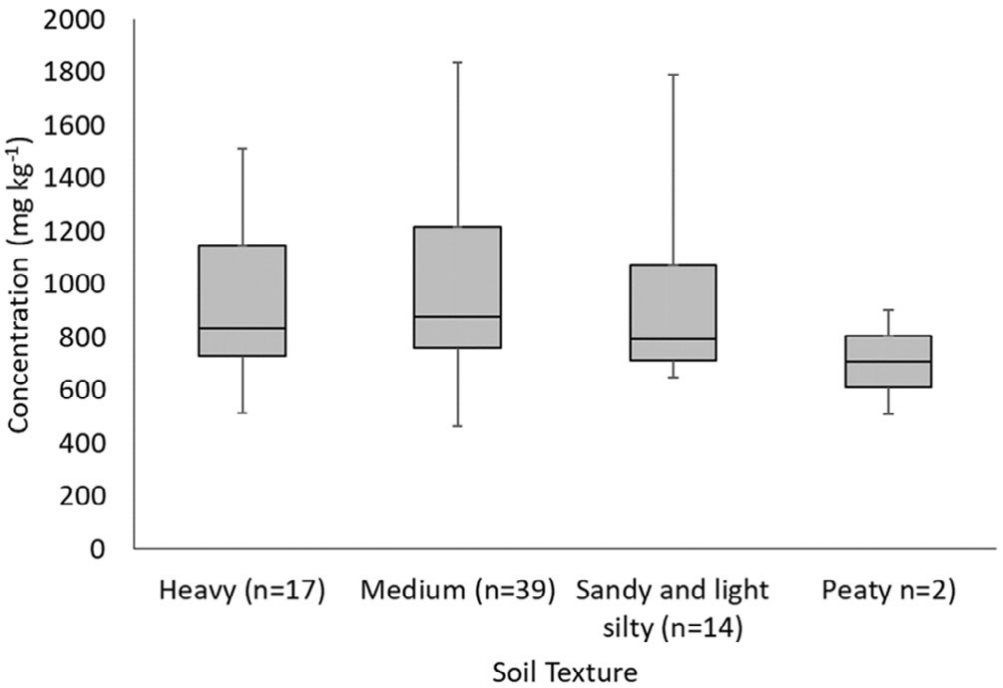
Box plot of channel bank profile middle section TP concentrations grouped by soil texture

No significant difference was found between channel bank TP concentration in banks grouped by geological parent material or WFD waterbody type. Comparisons of channel bank TP concentrations by soil texture also showed no statistically significant differences, apart from low values in the two sampled peaty bank profiles both of which were in the catchment of Semer Water lake in the upland Yorkshire Dales (Figure 6).

### 3.3 Inter‐ and intra‐site variability in TP concentrations in channel banks

**FIGURE 7:**
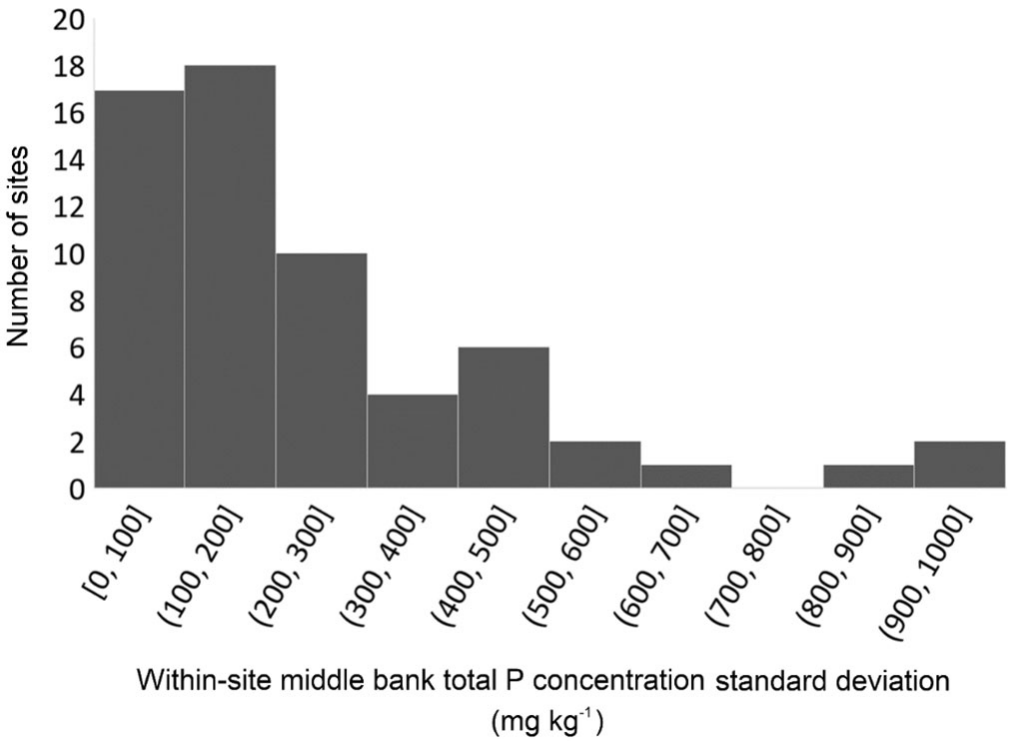
Histogram of intra‐site TP concentration standard deviations

When comparing the three channel bank profiles sampled within individual sites, a mean intra‐site standard deviation in TP content of 245 mg kg^−1^ was observed (Figure 7). An inter‐site standard deviation of 372 mg kg^−1^ was found when comparing the mean concentrations of the three replicates for each sampling site in the entire dataset. Therefore, local variability within sampling sites (a 25 m radius of located sampling point) makes up a significant proportion of the observed inter‐site variability.

### 3.4 National assessment of channel bank TP contributions to riverine total P loads

The original estimates of channel bank contributions to riverine total P loads in the SEPARATE tool assumed that the typical TP content of eroding channel banks across England is 550 mg kg^−1^ (Zhang et al., 2014). Targeted reconnaissance sampling across England in this study suggests that the previous use of this concentration could under‐estimate the contribution from channel banks to riverine total P loads (Table 1). Here, our new data suggest a median concentration of 873 mg kg^−1^ in channel banks, with a 25th percentile of 675 mg kg^−1^, and 75th percentile of 1159 mg kg^−1^, once outliers >2000 mg kg^−1^ were removed. Multiplying these updated concentration values and existing annual sediment loads from channel banks embedded in the SEPARATE tool, annual TP loads from this specific source were estimated. Assuming the same load contributions from the other sources included in the SEPARATE tool, all relative contributions were also recalculated. Taking published values as reference values, the underrepresentation of relative channel bank contributions to riverine total P loads across England could be between 23% and 111%, and most likely ~60%. Although channel banks are still rarely the dominant source of riverine total P loads in England, the revised TP contents increase the estimated number of inland WFD waterbodies in which bank erosion contributes >20% of the riverine total P load from 15 to 25 out of a total of 3792, with a corresponding range of 17–35 if the 25th and 75th percentile TP concentrations values are used. The total area of the WFD waterbodies in question thereby more than doubled from 144 to 303 km^2^ with a corresponding uncertainty range of 192–489 km^2^. On this basis, the proportion of the total inland surface waterbody area across England, wherein channel banks contribute >20% of the riverine total P load increased from 0.2% to 0.4%.

**TABLE 1:** WFD waterbody counts based on the relative contributions of riverine total P load originating from channel bank erosion

Channel bank contribution to total riverine P load	Using original data for channel bank TP content	Using new 25th percentile of TP content measured in channel banks	Using new median of TP content measured in channel banks	Using new 75th percentile of TP content measured in channel banks
(%)	(count)	(count)	(count)	(count)
5	3692	3652	3605	3545
10	63	86	112	147
15	16	24	38	37
20	6	13	12	28
30	5	5	10	18
40	1	3	5	4
50	3	2	1	3
60	2	3	3	1
70	3	2	2	4
90	1	2	4	5

## 4 DISCUSSION

Our TP concentrations (387 and 1626 mg kg^−1^, with a corresponding median of 873 mg kg^−1^) measured in the middle sections of channel bank profiles across England are similar to, but higher than those reported by the limited studies in the current international literature. Here, for example, a range of 249–452 mg kg^−1^ was reported by Thoma et al. (2005), compared with ranges of 303–555 mg kg^−1^ by Zaimes et al. (2008b), 246–349 mg kg^−1^ by Tufekcioglu (2010), 200–375 mg kg^−1^ (Purvis et al., 2016) and 400–1400 mg kg^−1^ by Kronvang et al. (2012).

TP concentrations in the channel bank profiles sampled across England were found to be primarily controlled by concentrations in the parent material rather than those in adjacent topsoils which have received inputs of organic matter and fertilizers. As a result, there is no significant correlation between channel bank and topsoil TP concentrations. Therefore, predicting local channel bank TP concentrations based upon readily accessible data on topsoil properties is not advisable. Whilst the estimation of channel bank TP concentrations using soil type or geology represents an approach with a greater chance of success, it was found that, in practice, neither factor was effective outside of isolated cases in our new dataset, such as Jurassic oordial ironstone in the River Nene catchment, or channel banks composed of peat. This is likely due to the high variability in channel bank TP content found within our sampling sites (a 25 m radius) which generated a standard deviation over half as high as the inter‐site standard deviation. As such, predicting channel bank TP concentrations at catchment scale in England is unlikely to be possible.

Significant spatial variability in channel bank TP concentrations has also been reported by existing work in streams in the same region with the same characteristics and land use (Granger et al., 2021; Miller et al., 2014; Purvis et al., 2016). Similarly, Zaimes et al. (2008b), Peacher et al. (2018) and Granger et al. (2021) also found that riparian land use was not a useful predictor of channel bank P concentrations. Porder and Ramachandran (2013) found that the P concentration of parent material explained 42% of the variance in total soil P concentration. The results of our study herein suggest that parent material has a much greater control on channel bank TP concentrations. Therefore, the use of a generic range of values generated from a database of channel bank TP concentrations is likely to be the best way to model channel bank contributions to total riverine P load.

There are implications arising from the estimated higher contributions from eroding channel banks to riverine total P loads in some WFD waterbodies across England. One implication is the increase in intrinsic TP concentrations in any catchment where only negligible pressures from human activities exist. This situation most likely occurs in pristine headwater catchments. The other implication is the generation of a ‘spatial mismatch’ for the efficacy of agricultural interventions for water quality protection between farm and catchment scale. In England, the current uptake of best management practices on farms for water quality protection is driven by a combination of regulation, incentivization and advice for win‐wins. Supplementary Table 2 summarizes a range of on‐farm interventions for water quality protection, with corresponding uptake rates for different soil and farm types, as well as the typical impacts of these measures for reducing TP losses to rivers at farm scale. These estimates are based on a combination of existing uptake surveys, and both empirical evidence and the elicitation of expert opinion regarding efficacy for reducing emissions of TP to water (Newell‐Price et al., 2011). Typical efficacy for reducing agricultural emissions of TP at farm scale ranges between 25% and 80% depending on the intervention in question. Our work herein on channel bank contributions to riverine total P loads suggests that in 17–35 WFD waterbodies across England, these efficacies for reducing TP losses to water at farm scale would be reduced at catchment scale by the contribution of bank‐derived TP. By way of comparison, with the updated bank erosion contribution to riverine total P loads based on using our new estimates of median TP content, the corresponding relative contributions of sewage treatment works (STWS), using the SEPARATE framework, exceed 50% in 744 waterbodies across England. For those WFD waterbodies with >10% of the riverine total P load originating from bank erosion (*n* = 75), 18 also have >10% originating from STWs. Here, the STW discharges of P in the SEPARATE framework were updated from those published in Zhang et al. (2014) using 2013–2016 data from the Environment Agency.

An additional consideration is that during periods of low flow, sediment is likely to accumulate on channel beds and in bed gravels where they exist (Lambert & Walling, 1988). During its storage on river beds, P can exchange between sediments and the overlying water column causing localized ecological harm (Ballantine et al., 2006; House & Denison, 1998; Jarvie et al., 2005; Rawlins, 2011). Sediment sourcing work has shown that a significant proportion of the sediment stored on river channel beds can originate from eroding channel banks (up to 19%—Collins & Walling, 2007; up to 100%—Pulley et al., 2019; up to 94%—Biddulph et al., 2017). By way of example, the sediment stored on the channel beds of lowland English catchments was found to have mean TP concentrations in the <63 μm fraction of 1355 mg kg^−1^ (River Frome), 1151 mg kg^−1^ (River Piddle), 1337 mg kg^−1^ (River Tern), 782 mg kg^−1^ (River Pang), 920 mg kg^−1^ (River Lambourn) by Collins et al. (2005). The median TP concentration of 873 mg kg^−1^ calculated for channel banks in this study indicates bank‐derived PP is likely to be a contributor to the PP present on the channel beds of many rivers in England.

Finally, whilst the work reported herein focusses on the SEPARATE tool, readers are reminded that such screening tools are used beyond the UK to assist water resource management. Here, examples include Ag‐PIE (Giupponi & Vladimirova, 2006), LENS (Loading Estimator of Nutrient Sources; Stainbrook et al., 2022) and work on integrated modelling of pollution risk and uncertainty surrounding the impacts of best management interventions (Brouwer & De Blois, 2008). Screening tools are more appropriate at strategic scales, than data‐hungry fully deterministic models, which can be applied at local scales where data availability is likely to be less problematic (Margane, 2003; Navulur & Engel, 1996). Pollutant screening tools often combine empirical and modelled data to undertake preliminary assessments of pollution source apportionment. The development and application of these tools generate generic needs, regardless of the pollutants or geography in question. These include careful consideration of the key pollutants and sources thereof that need to be included in any such tool, the availability of data to represent pollutant losses from individual sources and the ongoing need to update or improve the data used to represent individual sources. The latter need was the main driver for the work reported herein and whilst the focus was on channel TP, the remaining sources and their associated pollutants included in SEPARATE also warrant regular updates.

## 5 CONCLUSIONS

Whilst non‐agricultural point sources of P such as sewage treatment have been long recognized as a major cause of degraded water quality, diffuse non‐agricultural sources such as channel banks are often under considered in catchment management planning. The results of this study have shown that in most UK catchments banks are likely to contribute less than 5% of the riverine total P load. However, in 0.66% of catchments covering 0.2%–0.4% of the total inland surface waterbody area in England, they are estimated to contribute >20% of the total P load and in 5% of waterbodies, banks are estimated to contribute <5% of the P load. These contributions have the potential to generate a ‘spatial mismatch’ between the expected within‐stream outcomes of catchment management work targeting agricultural sources and the actual benefits delivered. Therefore, the potential for channel bank‐derived PP to reduce the benefits of management efforts should be considered when formulating a robust catchment management plan. It should be acknowledged, however, that bank‐derived PP is unlikely to be a major issue in most catchments outside of high‐risk geologies or where banks are heavily contaminated with P due to the legacy effect of intensive agricultural practices.

Predicting channel bank PP concentrations using readily available secondary data such as soil type, soil P concentration, or geology is unlikely to be successful. However, some specific geological units such as oordial ironstone or peat have a significant impact on channel bank PP concentrations. Instead, the use of a generic channel bank PP concentration of 873 mg kg^−1^ combined with modelled bank erosion rates is likely to be the optimal way to estimate the contribution of banks to riverine total P loads at national scale in England.

## Supporting information


**Data S1:** Supporting Information.

## Data Availability

The data that support the findings of this study are available from the corresponding author upon reasonable request.

## References

[hyp14785-bib-0001] Ballantine, D. J. , Walling, D. E. , Collins, A. L. , & Leeks, G. J. L. (2006). Phosphorus storage in fine channel bed sediments. Water, Air, and Soil Pollution: Focus, 6, 371–380. 10.1007/s11267-006-9029-2

[hyp14785-bib-0002] Beck, W. , Isenhart, T. , Moore, P. , Schilling, K. , Schultz, R. , & Tomer, M. (2018). Streambank alluvial unit contributions to suspended sediment and total phosphorus loads, Walnut Creek, Iowa, USA. Water, 10, 111. 10.3390/w10020111

[hyp14785-bib-0003] Biddulph, M. , Collins, A. L. , Foster, I. D. L. , & Holmes, N. (2017). The scale problem in tackling diffuse water pollution from agriculture: Insights from the Avon demonstration test catchment programme in England. River Research and Application., 33, 1527–1538. 10.1002/rra.3222

[hyp14785-bib-0004] Boorman, D. B. , Hollis, J. M. , & Lilly, A. (1995). Hydrology of soil types: A hydrologically based classification of the soils of the United Kingdom. Institute of Hydrology Report No. 126, Wallingford, UK, 137 pp.

[hyp14785-bib-0005] Brouwer, R. , & De Blois, C. (2008). Integrated modelling of risk and uncertainty underlying the cost and effectiveness of water quality measures. Environmental Modelling and Software, 23, 922–937.

[hyp14785-bib-0006] Collins, A. L. , & Anthony, S. G. (2008). Assessing the likelihood of catchments across England and Wales meeting ‘good ecological status’ due to sediment contributions from agricultural sources. Environmental Science and Policy, 11, 163–170. 10.1016/j.envsci.2007.07.008

[hyp14785-bib-0007] Collins, A. L. , Anthony, S. G. , Hawley, J. , & Turner, T. (2009a). The potential impact of projected change in farming by 2015 on the importance of the agricultural sector as a sediment source in England and Wales. Catena, 79, 243–250.

[hyp14785-bib-0008] Collins, A. L. , Anthony, S. G. , Hawley, J. , & Turner, T. (2009b). Predicting potential change in agricultural sediment inputs to rivers across England and Wales by 2015. Marine and Freshwater Research, 60, 626–637. 10.1071/MF08033

[hyp14785-bib-0009] Collins, A. L. , & Walling, D. E. (2007). Sources of fine sediment recovered from the channel bed of lowland groundwater fed catchments in the UK. Geomorphology, 88(1–2), 120–138. 10.1016/j.geomorph.2006.10.018

[hyp14785-bib-0010] Collins, A. L. , Walling, D. E. , & Leeks, G. J. L. (1997). Sediment sources in the upper Severn catchment: A fingerprinting approach. Hydrology and Earth System Sciences, 1, 509–521. 10.5194/hess-1-509-1997

[hyp14785-bib-0011] Collins, A. L. , Walling, D. E. , & Leeks, G. J. L. (2005). Storage of fine‐grained sediment and associated contaminants within the channels of lowland permeable catchments in the UK. Sediment Budgets 1 (Proceedings of symposium S1 held during the Seventh IAHS Scientific Assembly at Foz do Iguaçu, Brazil, April 2005). IAHS Publ. 291, 2005. 259.

[hyp14785-bib-0012] Collins, A. L. , Zhang, Y. , McChesney, D. , Walling, D. E. , Haley, S. M. , & Smith, P. (2012). Sediment source tracing in a lowland agricultural catchment in southern England using a modified procedure combining statistical analysis and numerical modelling. Science of the Total Environment, 414, 301–317. 10.1016/j.scitotenv.2011.10.062 22119027

[hyp14785-bib-0013] Cooper, D. M. , Naden, P. , Old, G. , & Laize, C. (2006). Development of guideline targets to support management of sediment inputs into aquatic ecosystems. Natural England Research Report NERR008, Sheffield, Natural England.

[hyp14785-bib-0014] De Rose, R. C. , Wilson, D. J. , Bartley, R. , & Wilkinson, S. N. (2005). Riverbank erosion and its importance to uncertainties in large scale sediment budgets. In D. E. Walling & J. A. Horowitz (Eds.), Proceedings of the international symposium on sediment budgets (S1) held during the seventh scientific assembly of the IAHS at Foz do Iguacu, Brazil, 3–9 April 2005, Sediment Budgets 1, IAHS Publ (Vol. 291, pp. 85–92). IAHS Press.

[hyp14785-bib-0015] Deuthmann, D. , Anthony, S. , Carvalho, L. , & Spears, B. (2009). A model‐based assessment of non‐compliance of phosphorus standards for lakes in England and Wales. International Journal of River Basin Management, 7, 197–207. 10.1080/15715124.2009.9635383

[hyp14785-bib-0016] Dixon, E. R. , Blakwell, M. S. A. , Dhanoa, M. S. , Berryman, Z. , de la Martinez, N. F. , Junquera, D. , Martinez, A. , Murray, P. J. , Kemp, H. F. , Meier‐Augenstein, W. , Duffy, A. , & Bol, R. (2010). Measurement at the field scale of soil 𝛿^13^C and 𝛿^15^ N under improved grassland. Rapid Communications in Mass Spectrometry, 24, 511–518. 10.1002/rcm.4345 20112268

[hyp14785-bib-0017] Evans, B. M. , Sheeder, S. A. , & Lehning, D. W. (2003). A spatial technique for estimating streambank erosion based on watershed characteristics. Journal of Spatial Hydrology, 3, 1–13.

[hyp14785-bib-0018] Evans, J. L. (2019). SMART: Sediment and mitigation options fo the river rother. Unpublished PhD Thesis, University of Northampton, UK.

[hyp14785-bib-0019] Fox, G. A. , Purvis, R. A. , & Penn, C. J. (2016). Streambanks: A net source of sediment and phosphorus to streams and rivers. Journal of Environmental Management, 181, 602–614. 10.1016/j.jenvman.2016.06.071 27429360

[hyp14785-bib-0020] George, T. S. , Giles, C. D. , Menezes‐Blackburn, D. , Condron, L. M. , Gama‐Rodrigues, A. C. , Jaisi, D. , Lang, F. , Neal, A. L. , Stutter, M. I. , Almeida, D. S. , Bol, R. , Cabugao, K. G. , Celi, L. , Cotner, J. B. , Feng, G. , Goll, D. S. , Hallama, M. , Krueger, J. , Plassard, C. , … Haygarth, P. M. (2017). Organic phosphorus in the terrestrial environment: A perspective on the state of the art and future priorities. Plant and Soil, 427, 191–208. 10.1007/s11104-017-3391-x

[hyp14785-bib-0083] Giupponi, C., & Vladimirova, I. (2006). Ag‐PIE: A GIS‐Based Screening model for assessing agricultural pressures and impacts on water quality on a European Scale. Science of the Total Environment, 359, 57–75. 10.1016/j.scitotenv.2005.07.013 16181658

[hyp14785-bib-0021] Glavan, M. , White, S. M. , & Holman, I. P. (2012). Water quality targets and maintenance of valued landscape character: Experience in the axe catchment, UK. Journal of Environmental Management, 103, 142–153. 10.1016/j.jenvman.2012.03.009 22475720

[hyp14785-bib-0023] Gooday, R. D. , Anthony, S. G. , Durrant, C. , Harris, D. , Lee, D. , Metcalfe, P. , Newell‐Price, P. , & Turner, A. (2015). Farmscoper extension report for Defra project SCF0104.

[hyp14785-bib-0024] Granger, S. J. , Harris, P. , Upadhayay, H. R. , Sint, H. , Pulley, S. , Stone, M. , Krishnappan, B. G. , & Collins, A. L. (2021). Novel approaches to investigating spatial variability in channel bank total phosphorus at the catchment scale. Catena, 202, 105223. 10.1016/j.catena.2021.105223

[hyp14785-bib-0025] Guo, J. , & Julien, Y. P. (2005). Shear stress in smooth rectangular open‐channel flows. Journal of Hydraulic Engineering ASCE, 131, 30–37.

[hyp14785-bib-0026] Gustard, A. , Bullock, A. , & Dixon, J. M. (1992). Low flow estimation in the United Kingdom. Institute of Hydrology Report 108, Wallingford, UK.

[hyp14785-bib-0027] Haygarth, P. M. , ApSimon, H. , Betson, M. , Harris, D. , Hodgkinson, R. , & Withers, P. J. A. (2009). Mitigating diffuse phosphorus transfer from agriculture according to cost and efficiency. Journal of Environmental Quality, 38, 2012–2022. 10.2134/jeq2008.0102 19704144

[hyp14785-bib-0028] Heathwaite, A. L. , Dils, R. M. , Liu, S. , Carvalho, L. , Brazier, R. E. , Pope, L. , Hughes, M. , Phillips, G. , & May, L. (2005). A tiered risk‐based approach for predicting diffuse and point source phosphorus losses in agricultural areas. Science of the Total Environment, 344, 225–239. 10.1016/j.scitotenv.2005.02.034 15907520

[hyp14785-bib-0029] Horowitz, A. (1991). A primer on sediment‐trace element chemistry (2nd ed., p. 136). Lewis Publishing Co.

[hyp14785-bib-0030] House, W. A. , & Denison, F. H. (1998). Phosphorus dynamics in a lowland river. Water Research, 32, 1819–1830. 10.1016/j.ecoleng.2005.06.013

[hyp14785-bib-0031] Howe, E. , Winchell, M. , Meals, D. , Folle, S. , Moore, J. , Braun, D. , DeLeo, C. , Budreski, K. , & Schiff, R. (2011). Identification of critical source areas of phosphorus within the Vermont sector of the Missisquoi Bay basin. Stone Environmental Inc: Prepared for Lake Champlain Basin Program, Grand Isle, VT.

[hyp14785-bib-0032] Imeson, A. C. , Vis, M. , & Duysings, J. J. H. M. (1984). Surface and subsurface sources of suspended solids in forested drainage basins in the Keuper region of Luxembourg. In T. P. Burt & D. E. Walling (Eds.), Catchment experiments in fluvial geomorphology (pp. 219–234). GeoBooks.

[hyp14785-bib-0033] Janes, V. J. J. , Nicholas, A. P. , Collins, A. L. , & Quine, T. A. (2017). Analysis of fundamental physical factors influencing channel bank erosion: Results for contrasting catchments in England and Wales. Environ Earth Science, 76, 307. 10.1007/s12665-017-6593-x

[hyp14785-bib-0034] Jarvie, H. P. , Jurgens, M. D. , Williams, R. J. , Neal, C. , Davies, J. J. L. , Barrett, C. , & White, J. (2005). Role of river bed sediments as sources and sinks of phosphorus across two major eutrophic UK river basins: The Hampshire Avon and Herefordshire Wye. Journal of Hydrology, 304, 51–74. 10.1016/j.jhydrol.2004.10.002

[hyp14785-bib-0035] Jones, J. I. , Murphy, J. F. , Collins, A. L. , Sear, D. A. , & Naden, P. S. (2012). The impact of fine sediment on macro‐invertebrates. River Research and Applications, 28, 1055–1071. 10.1002/rra.1516

[hyp14785-bib-0036] Julian, J. P. , & Torres, R. (2006). Hydraulic erosion of cohesive riverbanks. Geomorphology, 76, 193–206. 10.1016/j.geomorph.2005.11.003

[hyp14785-bib-0037] Kronvang, B. , Andersen, H. E. , Larsen, S. E. , & Audet, J. (2013). Importance of bank erosion for sediment input, storages and export at the catchment scale. Journal of Soils and Sediments, 13, 230–241. 10.1007/s11368-012-0597-7

[hyp14785-bib-0038] Kronvang, B. , Audet, J. , Baattrup‐Pedersen, A. , Jensen, H. S. , & Larsen, S. E. (2012). Phosphorus loads to surface water from bank erosion in a Danish lowland river basin. Journal of Environmental Quality, 41, 304–313. 10.2134/jeq2010.0434 22370392

[hyp14785-bib-0039] Kronvang, B. , Laubel, A. , & Grant, R. (1997). Suspended sediment and particulate phosphorus transport and delivery pathways in an arable catchment, Gelbæk Stream. Denmark. Hydrological Processes, 11, 627–642. 10.1002/(SICI)1099-1085(199705)11:6<627::AID-HYP481>3.0.CO;2-E

[hyp14785-bib-0040] Kronvang, B. , Vagstad, N. , Behrendt, H. , Bogestrand, J. , & Larsen, S. E. (2007). Phosphorus losses at the catchment scale within Europe: An overview. Soil Use and Management, 23, 104–116. 10.1111/j.1475-2743.2007.00113.x

[hyp14785-bib-0082] Lambert, C. P., & Walling, D. E. (1988). Measurement of channel storage of suspended sediment in a gravel‐bed river. CATENA, 15(1), 65–80. 10.1016/0341-8162(88)90017-3

[hyp14785-bib-0041] Laubel, A. , Kronvang, B. , Hald, A. B. , & Jensen, C. (2003). Hydromorphological and biological factors influencing sediment and phosphorus loss via bank erosion in small lowland rural streams in Denmark. Hydrological Processes, 17, 3443–3463. 10.1002/hyp.1302

[hyp14785-bib-0042] Lloyd, C. E. M. , Johnes, P. J. , Freer, J. E. , Carswell, A. M. , Jones, J. I. , Stirling, M. W. , Hodgkinson, R. A. , Richmond, C. , & Collins, A. L. (2019). Determining the sources of nutrient flux to water in headwater catchments: Examining the speciation balance to inform the targeting of mitigation measures. Science of the Total Environment, 648, 1179–1200. 10.1016/j.scitotenv.2018.08.190 30340264

[hyp14785-bib-0043] Lu, S. , Kronvang, B. , Audet, J. , Trolle, D. , Andersen, H. E. , Thodsen, H. , & van Griensven, A. (2015). Modelling sediment and total phosphorus export from a lowland catchment: Comparing sediment routing methods. Hydrological Processes, 29, 280–294. 10.1002/hyp.10149

[hyp14785-bib-0044] Margane, A. (2003). Management, protection and sustainable use of groundwater and soil resources in the Arab region: guideline for groundwater vulnerability mapping and risk assessment for the sustainability of groundwater resources to contamination. Project N‐1996.2189.7—Arab Centre for the Study of the Arid Zones and Dry Lands (ACSAD) and Federal Institute for Geosciences and Natural Resources (BGR). Damascus (2003), 53 pp.

[hyp14785-bib-0045] McDowell, R. W. , Dils, R. M. , Collins, A. L. , Flahive, K. A. , Sharpley, A. N. , & Quinn, J. (2016). A review of the policies and implementation of practices to decrease water quality impairment by phosphorus in New Zealand, the UK, and the US. Nutrient Cycling in Agroecosystems, 104, 289–305. 10.1007/s10705-015-9727-0

[hyp14785-bib-0046] Meybeck, M. , & Helmer, R. (1989). The quality of rivers ‐ from pristine stage to global pollution. Global and Planetary Change, 75, 283–309. 10.1016/0921-8181(89)90007-6

[hyp14785-bib-0047] Miller, R. B. , Fox, G. A. , Penn, C. J. , Wilson, S. , Parnell, A. , Purvis, R. A. , & Criswell, K. (2014). Estimating sediment and phosphorus loads from streambanks with and without riparian protection. Agriculture, Ecosystems & Environment, 189, 70–81. 10.1016/j.agee.2014.03.016

[hyp14785-bib-0048] Morse, G. K. , Laster, J. N. , & Perry, R. (1993). The economic and environmental impact of phosphorus removal from wastewater in the European Community. Selper Publications.

[hyp14785-bib-0049] Murphy, J. , & Riley, J. P. (1962). A modified single solution method for the determination of phosphate in natural waters. Analytica Chimica Acta, 27, 31–36. 10.1016/S0003-2670(00)88444-5

[hyp14785-bib-0050] Navulur, K. C. S. , & Engel, B. A. (1996). Predicting spatial distributions of vulnerability of Indiana State aquifer systems to nitrate leaching using a GIS. Third International Conference/Workshop on Integrating GIS and Environmental Modelling, Santa Fe, New Mexico, USA, 21–25 January (1996). http://www.ncgia.ucsb.edu/conf/SANTA_FE_CD-ROM/sf_papers/navulur_kumar/my_paper.html

[hyp14785-bib-0051] Neal, C. W. M. , & Andera, A. M. (2015). Suspended sediment supply dominated by bank erosion in a low‐gradient agricultural watershed, wildcat Slough, fisher, Illinois, United States. Journal of Soil Water Conservation, 70, 145–155. 10.2489/jswc.70.3.145

[hyp14785-bib-0052] Newell‐Price, J. P. , Harris, D. , Taylor, M. , Williams, J. R. , Anthony, S. G. , Chadwick, D. R. , Chambers, B. J. , Duethmann, D. , Gooday, R. D. , Lord, E. I. , Chadwick, D. R. , Misselbrook, T. H. , & Smith, K. A. (2011). User Manual‐‘ALL’. An Inventory of Methods and Guide to their Effects on Diffuse Water Pollution, Greenhouse Gas Emissions and Ammonia Emissions from Agriculture—User Guide. Defra project WQ0106(5). Final Report.

[hyp14785-bib-0053] Owens, P. N. , Walling, D. E. , & Leeks, G. J. L. (2000). Tracing fluvial suspended sediment sources in the catchment of the river tweed, Scotland, using composite fingerprints and a numerical mixing model. In I. D. L. Foster (Ed.), Tracers in geomorphology (pp. 291–308). John Wiley & Sons Ltd.

[hyp14785-bib-0054] Peacher, R. D. , Lerch, R. N. , Schultz, R. C. , Willett, C. D. , & Isenhart, T. M. (2018). Factors controlling streambank erosion and phosphorus loss in claypan watersheds. Journal of Soil and Water Conservation, 73, 189–199. 10.2489/jswc.73.2.189

[hyp14785-bib-0055] Porder, S. , & Ramachandran, S. (2013). The phosphorus concentration of common rocks—A potential driver of ecosystem P status. Plant and Soil, 367, 41–55. 10.1007/s11104-012-1490-2

[hyp14785-bib-0056] Pulley, S. , Goubet, A. , Moser, I. , Browning, S. , & Collins, A. L. (2019). The sources and dynamics of fine‐grained sediment degrading the freshwater pearl mussel (*Margaritifera margaritifera*) beds of the river Torridge, Devon, UK. Science of the Total Environment, 657, 420–434. 10.1016/j.scitotenv.2018.11.401 30550906 PMC6372835

[hyp14785-bib-0057] Purvis, R. A. , Fox, G. A. , Penn, C. J. , Storm, D. E. , & Parnell, A. (2016). Estimating streambank phosphorus loads at the watershed scale with uncertainty analysis approach. Journal of Hydrologic Engineering, 21, 12. 10.1061/(asce)he.1943-5584.0001402

[hyp14785-bib-0058] Radcliffe, D. E. , Freer, J. , & Schoumans, O. (2009). Diffuse phosphorus models in the United States and Europe: Their usages, scales, and uncertainties. Journal of Environmental Quality, 38(5), 1956–1967. 10.2134/jeq2008.0060 19704139

[hyp14785-bib-0059] Rawlins, B. G. (2011). Controls on the phosphorus content of fine stream bed sediments in agricultural headwater catchments at the landscape‐scale. Agriculture, Ecosystems and Environment, 144, 352–363. 10.1016/j.agee.2011.10.002

[hyp14785-bib-0060] Rockström, J. , Steffen, W. , Noone, K. , Persson, Å. , Chapin, F. S. , Lambin, E. , Lenton, T. M. , Scheffer, M. , Folke, C. , Schellnhuber, H. , Nykvist, B. , De Wit, C. A. , Hughes, T. , van der Leeuw, S. , Rodhe, H. , Sörlin, S. , Snyder, P. K. , Costanza, R. , Svedin, U. , … Foley, J. (2009). Planetary boundaries: Exploring the safe operating space for humanity. Ecology and Society, 14, 32. 10.5751/ES-03180-140232 19779433

[hyp14785-bib-0061] Rode, M. , op de Hipt, F. , Collins, A. L. , Zhang, Y. S. , Theuring, P. , Schkade, U. K. , & Diekkruger, B. (2018). Subsurface sources contribute substantially to fine‐grained suspended sediment transported in a tropical West African watershed in Burkina Faso. Land Degradation and Development, 29, 4092–4105. 10.1002/ldr.3165

[hyp14785-bib-0062] Roseboom, D. P. (1987). Case studies of stream and river restoration. in: Urbana‐Champaign, U.o.I.a. (Ed.), Management of the Illinois River System: The 1990's and Beyond: Proceedings: a Governor's Conference on April 1–3, 1987 at Peoria, Illinois for Citizens, Organizations, Industry, and Government Representatives and Resources Management Professionals. Water Resources Center, University of Illinois, pp. 184–194.

[hyp14785-bib-0063] Royer, T. V. , David, M. B. , & Gentry, L. E. (2006). Timing of riverine export of nitrate and phosphorus from agricultural watersheds in Illinois: Implications for reducing nutrient loading to the Mississippi River. Environmental Science & Technology, 40, 4126–4131. 10.1021/es052573n 16856726

[hyp14785-bib-0064] Sekely, A. C. , Mulla, D. J. , & Bauer, D. W. (2002). Streambank slumping and its contribution to the phosphorus and suspended sediment loads of the Blue Earth River, Minnesota. Journal of Soil and Water Conservation, 57, 243–250.

[hyp14785-bib-0065] Smith, B. F. L. , & Bain, D. C. (1982). A sodium hydroxide fusion method for the determination of total phosphate in soils. Communications in Soil Science and Plant Analysis, 13(3), 185–190. 10.1080/00103628209367257

[hyp14785-bib-0066] Stainbrook, K. , Ross, C. , Davis, C. , & Townley, L. (2022). Developing a watershed screening tool to estimate relative contribution of phosphorus to guide management planning. Journal of Environmental Management, 312, 114937. 35398696 10.1016/j.jenvman.2022.114937

[hyp14785-bib-0081] Stone, M., & English, M. C. (1993). Geochemical composition, phosphorus speciation and mass transport of fine‐grained sediment in two Lake Erie tributaries. Hydrobiologia, 253, 17–29. 10.1007/BF00050719

[hyp14785-bib-0067] Stone, M. , & Mudroch, A. (1989). The effect of particle‐size, chemistry and mineralogy of river sediments on phosphate adsorption. Environmental Science & Technology Letters, 10, 501–510. 10.1080/09593338909384766

[hyp14785-bib-0068] Taylor, A. W. , & Kunishi, H. M. (1971). Phosphate equilibria on stream sediment and soil in a watershed draining an agricultural region. Journal of Agricultural and Food Chemistry, 19, 827–831. 10.1021/jf60177a061

[hyp14785-bib-0069] Thoma, D. P. , Gupta, S. C. , Bauer, M. E. , & Kirchoff, C. E. (2005). Airborne laser scanning for river bank erosion assessment. Remote Sensing of the Environment, 95, 493–501. 10.1016/j.rse.2005.01.012

[hyp14785-bib-0070] Tufekcioglu, M. (2010). Stream bank soil and phosphorus losses within grazed pasture stream reaches in the Rathbun watershed in southern Iowa. PhD thesis. Iowa State University.

[hyp14785-bib-0071] Tye, A. M. , Rawlins, B. G. , Rushton, J. C. , & Price, R. (2016). Understanding the controls on sediment‐P interactions and dynamics along a non‐tidal river system in a rural–urban catchment: The River Nene. Applied Geochemistry, 66, 219–233. 10.1016/j.apgeochem.2015.12.014

[hyp14785-bib-0072] Walling, D. E. , & Collins, A. L. (2005). Suspended sediment sources in British rivers. In: Sediment budgets 1 International Association of Hydrological Sciences Publication No. 291, Wallingford, pp. 123–133.

[hyp14785-bib-0073] Walling, D. E. , Collins, A. L. , & Stroud, R. (2008). Tracing suspended sediment and particulate phosphorus sources in catchments. Journal of Hydrology, 350, 274–289. 10.1016/j.jhydrol.2007.10.047

[hyp14785-bib-0074] Walling, D. E. , Owen, P. , & Leeks, G. J. L. (1999). Fingerprinting suspended sediment sources in the catchment of the river Ouse, Yorkshire. Hydrological Processes, 13, 977–992. 10.1002/(SICI)1099-1085(199905)13:7<955::AID-HYP784>3.0.CO;2-G

[hyp14785-bib-0075] Walling, D. E. , & Webb, B. W. (1987). Suspended load in gravel‐bed rivers: UK experience. In C. R. Thorne , J. C. Bathurst , & R. D. Hey (Eds.), Sediment transport in gravel‐bed rivers (pp. 691–723). Wiley.

[hyp14785-bib-0076] Walling, D. E. , Webb, B. W. , & Shanahan, J. (2008). Investigations into the use of critical sediment yields for assessing and managing fine sediment inputs into freshwater ecosystems. Natural England Research Report NERR007, Natural England, Sheffield.

[hyp14785-bib-0077] Wilkinson, S. N. , Olley, J. M. , Prosser, I. P. , & Read, A. M. (2005). Targeting erosion control in large river systems using spatially distributed sediment budgets, geomorphological processes and human impacts in river basins. In International Association of Hydrological Sciences Publication No. 299 (pp. 56–64). IAHS Press.

[hyp14785-bib-0078] Zaimes, G. N. , Schultz, R. C. , & Isenhart, T. M. (2008a). Streambank soil and phosphorus losses under different riparian land‐uses in Iowa. Journal of the American Water Resources Association, 44, 935–947. 10.1111/j.1752-1688.2008.00210.x

[hyp14785-bib-0079] Zaimes, G. N. , Schultz, R. C. , & Isenhart, T. M. (2008b). Total phosphorus concentrations and compaction in riparian areas under different riparian land‐uses of Iowa. Agriculture, Ecosystems & Environment, 127, 22–30. 10.1016/j.agee.2008.02.008

[hyp14785-bib-0080] Zhang, Y. , Collins, A. L. , Murdoch, N. , Lee, D. , & Naden, P. S. (2014). Cross sector contributions to river pollution in England and Wales: Updating waterbody scale information to support policy delivery for the water framework directive. Environmental Science & Policy, 42, 16–32. 10.1016/j.envsci.2014.04.010

